# Rapid Urban Malaria Appraisal (RUMA) II: Epidemiology of urban malaria in Dar es Salaam (Tanzania)

**DOI:** 10.1186/1475-2875-5-28

**Published:** 2006-04-04

**Authors:** Shr-Jie Wang, Christian Lengeler, Deodatus Mtasiwa, Thomas Mshana, Lusinge Manane, Godson Maro, Marcel Tanner

**Affiliations:** 1 Swiss Tropical Institute, P.O. Box, CH-4002 Basel, Switzerland; 2 The Dar es Salaam Regional/City Medical Office of Health, P.O. Box 9084, Dar es Salaam, Tanzania; 3 Medical Laboratory Scientists Association of Tanzania, P.O. Box 65094, Dar es Salaam, Tanzania; 4 The Muhimbili University College of Health Sciences, P.O. Box 35091, Dar es Salaam, Tanzania

## Abstract

**Background:**

The thinking behind malaria research and control strategies stems largely from experience gained in rural areas and needs to be adapted to the urban environment.

**Methods:**

A rapid assessment of urban malaria was conducted in Dar es Salaam in June-August, 2003 using a standard Rapid Urban Malaria Appraisal (RUMA) methodology. This study was part of a multi-site study in sub-Saharan Africa supported by the Roll Back Malaria Partnership.

**Results:**

Overall, around one million cases of malaria are reported every year by health facilities. However, school surveys in Dar es Salaam during a dry spell in 2003 showed that the prevalence of malaria parasites was low: 0.8%, 1.4%, 2.7% and 3.7% in the centre, intermediate, periphery and surrounding rural areas, respectively. Health facilities surveys showed that only 37/717 (5.2%) of presenting fever cases and 22/781 (2.8%) of non-fever cases were positive by blood slide. As a result, malaria-attributable fractions for fever episodes were low in all age groups and there was an important over-reporting of malaria cases. Increased malarial infection rates were seen in persons who travelled to rural areas within the past three months. A remarkably high coverage of insecticide-treated nets and a corresponding reduction in malarial infection risk were found.

**Conclusion:**

The number of clinical malaria cases was much lower than routine reporting suggested. Improved malaria diagnosis and re-defined clinical guidelines are urgently required to avoid over-treatment with antimalarials.

## Introduction

Rapid urbanization brings about major changes in ecology, social structure and disease patterns in sub-Saharan Africa. It is estimated that 300 million people currently live in urban areas in Africa and two-thirds of them are at risk of malaria [1]. There is a lack of understanding of the complex interactions between human social structure, the environment and malaria infections [2-4]

Malaria research and control efforts in Tanzania began in the late 1890s, both in urban and in rural areas [5,6]. In the 1970s the malaria problem emerged again on a large scale in Dar es Salaam, mainly because of the deterioration of the health care system. In 2000, 33% of the population in Tanzania lived in urban areas [7] and urban poverty was widespread and increasing. More attention is now being devoted again to urban malaria, as uncontrolled urban population growth calls for upscaled and adapted strategies [8,9].

There are only a few papers concerning malaria epidemiology in Dar es Salaam. Okeahialam *et al. *[10] examined 218 hospital inpatients and 422 outpatients throughout 1971 and found that 20% of fever cases had malaria parasitaemia. Mkawagile [11] reported that during the heavy rainy season in 1981, about 47.6% of adult outpatients attending Mwananyamala hospital with typical malaria symptoms had parasitaemia; among all outpatients the parasitaemia prevalence was only 27%. Makani [12] noted that 87% of patients who received antimalarial treatment in Muhimbili National Hospital for presumed severe malaria did not have detectable parasitaemia. In that situation, over-diagnosis of cerebral malaria in patients with neurological dysfunction resulted in over-treatment of malaria and a neglect of other potentially life-threatening conditions. Yamagata [8] reported that the malaria prevalence rates among schoolchildren in the central, intermediate and peripheral zones of Dar es Salaam in 1988 were roughly 6%, 28–41% and 68–74%. Following the implementation of the first Urban Malaria Control Project (UMCP) during the period 1988–1994 these rates decreased to 3–10%, 10–25% and 21–46%.

A standard study protocol for Rapid Urban Malaria Appraisal (RUMA) was developed in June, 2002, based on a WHO proposal and an Environmental Health Project draft protocol [13,14]. RUMAs were commissioned by the Roll Back Malaria Partnership for three Francophone countries (Côte d'Ivoire, Burkina Faso and Benin) and one Anglophone country (Tanzania). Each of the four assessments provided the following information: an overview of the urbanization history, an estimate of the fraction of fevers attributable to malaria, parasite rates for different city areas, an outline of health care services and highlights of the *lessons learned *[15]. The aim of the present study was to compile a minimum dataset to identify key malaria issues affecting Dar es Salaam within a 6–10 weeks time frame. In addition, malaria vulnerability in relation to urban agriculture, socio-economic factors and rural exposure were assessed.

## Methods

### Study site and sample selection

**Figure 1:**
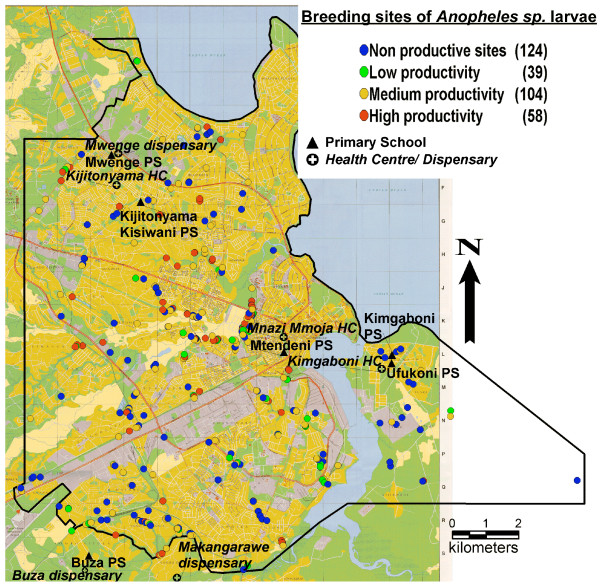
Map of selected schools and health facilities in relation to *Anopheles sp*. breeding sites. Source: Adapted from Sattler *et al. *[16]. p/s = primary school. HC = health center.

Dar es Salaam is situated between latitude 6.0°–7.5°S and longitude 39.0°–39.6°E. It had 2,500,000 inhabitants in 2002 (a density of 1,800 per sq. km) [16]. The municipality is divided into three districts: Ilala, Kinondoni and Temeke. To study the heterogeneity of malaria risk, Dar es Salaam was divided into four zones: centre, intermediate, periphery and surrounding rural areas. The zones were defined on the basis of city characteristics and the potential malaria risk indicated by an existing *Anopheles *breeding site maps (Figure 1) [8,16]. Due to the time constraints of a RUMA, only one or two representative health facilities and one or two representative schools in each zone could be selected (two units were selected when the target sample size could not be reached in a single unit).

#### Centre

Mtendeni primary school and Mnazi Mmoja Health Centre are located in Ilala District facing the harbour and Mbagala Creek (Figures 1). It is a trader-dominated commercial centre of the inner city. They are located approximately 1–2 km from Msimbazi Valley where *Anopheles sp. *breeding sites are numerous [16].

#### Intermediate zone

Mwenge primary school, Kijitonyama Kisiwani primary school, Mwenge dispensary and Kijitonyama dispensary are located in Kijitonyama Ward in Kinondoni District in a middle class suburb (Figure 1). There are only few breeding sites in this area, apart from one with high productivity near Kijitonyama Kisiwani primary school (1 km away). Mwenge primary school is far from the identified breeding sites.

#### Periphery

in Temeke District, Ufukoni primary schools and Kigamboni Health Centre in Kigamboni Ward were chosen (Figure 1). Kigamboni Ward is a new peri-urban low-income suburb south of Dar es Salaam associated with a medium level of mosquito breeding sites.

#### Rural zone

Buza primary school, Buza dispensary and Makangarawe dispensary are located at the emerging urban-rural interface on the hill beside Buza Forest in Temeke District (Figure 1). Most children were from Makangarawe and Yombo Vituka Wards. The surroundings of Yombo Vituka consist of several large open fields, and there is a high risk of malaria transmission according to the available *Anopheles sp. *breeding site map. The area is favourable to newcomers [17] and there is a high proportion of low-income households.

### RUMA Methodology

Details for the RUMA methods are given in an overview publication [15]. Briefly, the following components were included.

#### Review of literature and collection of health statistics

Published information on malaria epidemiology was reviewed systematically through a literature search in the main bibliographic databases (PUBMED and EMBASE), through scanning reference lists and through contacting relevant experts, nationally and internationally. Unpublished data were obtained from the Dar es Salaam City Medical Office of Health (CMOH) and Dar es Salaam Urban Health Project (DUHP). Demographic data and malaria reports were collected from the three urban District Medical Offices (DMO) of Iala, Kinondoni and Temeke, the CMOH, from the Ministry of Health (MOH) and from the Population and Housing Census Bureau of Tanzania.

#### School parasitaemia surveys

A cross-sectional school parasitaemia survey was conducted during the dry season (July 20–30, 2003). Roughly 200 students aged 6–12 years were recruited in each zone, with the exception of Ufukoni primary school (389 children). Consent forms were given to the guardians or household heads. Only children who returned the consent forms had an axillary temperature measurement and a blood sample taken. Both thin and thick films were taken, stained with Giemsa and examined in the laboratory of the main municipal hospital (Amana hospital). Parasite density was defined as the number of parasites per 200 white blood cells. The children were interviewed with the assistance of school teachers regarding their family situation and malaria infection history.

#### Health facility fever surveys

The health facility surveys aimed at determining the malaria prevalence among fever cases and the fraction of malaria attributable fevers [18]. The surveys were carried out between July 16 and August 15, 2003. Two hundreds fever cases and 200 non-fever controls were recruited from one to two clinics located in each area. About 50% of the sample population was aged ≤ five years. Outpatients with a history of fever (past 36 hours) or with a measured temperature of ≥ 37.5°C were defined as cases. After being recruited and giving informed consent, each patient had an axillary temperature measurement and a blood film taken. An armpit temperature reading is usually 0.3°C to 0.6°C lower than an oral temperature reading and therefore 0.5°C was added to the reading, giving the final "measured" temperature for that individual. A control group was recruited from another department of the same hospital without current or past fever, matched by age and by residency with the case group. Exclusion criteria were: patients with signs of severe disease, patients returning to the health facility for follow-up visits, non-permanent town residents (less than six months per year). Patients were further interviewed concerning their socio-economic status, ITN usage, travel and malaria treatment history and health care seeking strategy.

The odds ratio (OR) that was calculated is the ratio of the odds of having parasitaemia in fever cases over non-fever controls. The formula for the fraction of fever episodes attributable to malaria parasites is: (1-1/Odds Ratio)*P, with P being the proportion of fever episodes in which the subjects also had malaria parasites [18].

In order to evaluate the quality of the slide reading, 200 slides were re-examined at the Ifakara Health Research and Development Centre in Tanzania and then a second time at the Swiss Tropical Institute (STI) in Basel, Switzerland. Quality control readings agreed for 197 slides. The sensitivity, specificity, and accuracy rates of slide readings were: 83.3%, 99.0% and 98.5%.

#### Mapping activities

Malaria risk mapping: the breeding sites mapping was done in conjunction with another project. The mapping was carried out in 151 km^2 ^of inner Dar es Salaam from March 1 to May 29, 2003. A detailed review of habitat characterization and spatial distribution of *Anopheles sp*. larvae is already published [16].

Mapping of health facilities: it was carried out within three weeks by three geography students of the University of Dar es Salaam using a global positioning system (GPS). With the guidance of public and private health facilities supervisors and the Geographic Information System (GIS) unit of CMOH, all existing health facilities were visited and the locations were recorded.

#### Brief description of the health care system

The quality of health services determines the effectiveness of malaria case management. Three documents from the DUHP were used as basis for a brief evaluation of the health care system in Dar es Salaam [14,19,20].

### Statistical methods

The data were double-entered and validated in EpiInfo 6.04 (CDC Atlanta, USA, 2001). Data analysis was carried out in Stata 8 (Stata Corp. Texas, USA, 2003). The X^2 ^test was applied to assess associations between categorical variables. Logistic regression was performed to assess the association between binary outcomes (mainly parasitaemia) and explanatory variables.

### Ethics

Ethical clearance was granted by the Medical Coordinating Committee of the National Institute for Medical Research, Tanzania. All patients gave written informed consent for the study. A prescription of sulfadoxine/pyrimethamine or amodiaquine and paracetamol was paid for if the patients presented a fever sign.

## Results

Between 1961–2004, 29 papers were found concerning malaria in Dar es Salaam. Only three papers and one document were related to clinical malaria and malaria endemicity.

### Brief description of the health care system

**Figure 2:**
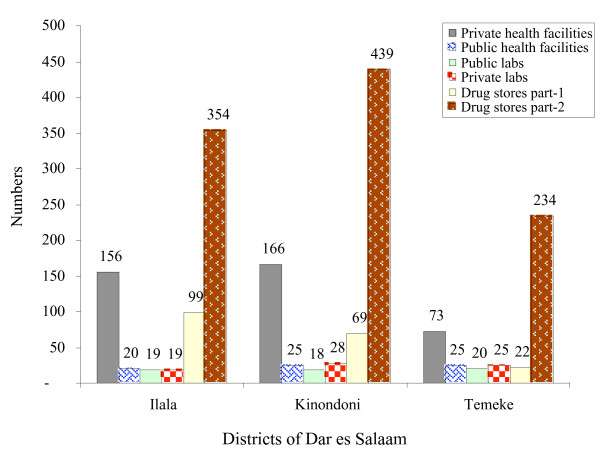
Distribution of public/private health facilities in Dar es Salaam. Drug stores part-1=Prescription pharmacies. Drug stores part-2=Non-prescription drug outlets.

There are five levels in the public health care system in Tanzania, but only three levels exist under the Dar es Salaam CMOH: districts (each with a municipal hospital), divisions (each with a health centre) and wards (with dispensaries and affiliated clinics). From October, 1990 to the end of 2002, the Dar es Salaam Health Project, supported by the Swiss Agency for Development and Cooperation and the Swiss Tropical Institute (STI), assisted with the rehabilitation of health facilities, the process of decentralization of decision-making at all levels and improvements of the drug and medical supply management [19]. In total 64 public (three district hospitals, five health centres and 56 dispensaries) and 395 private health facilities were registered in July, 2003 by the CMOH [21]. In addition, three hospitals in Dar es Salaam were under the prison, police or military authority (Figure 2). The privatization of health services is currently booming, but the CMOH supervision and inspection of private health facilities is loose.

The public health services were fairly well distributed by ward. Daily attendance at the public health facilities were 2,387, 3,361 and 2,873 in Ilala, Kinondoni and Temeke, respectively. The private health service providers were distributed heterogeneously (156 in Ilala, 166 in Kinondoni and only 73 in Temeke), and the majority of these facilities were located in the better-off inner city – obviously depending on the cash availability of patients. Voluntary services were well represented in the deprived areas at the city fringes. On average, 70% of the population lived within 5 km of a health facility [22].

The pharmacy board of Tanzania has implemented an official drug registration procedure in the year 2000. In 2003, there were 190 prescription pharmacies (drug stores part-1) or 99, 69 and 22 in Temeke, Kinondoni and Ilala, respectively. Further, there were 1,027 non-prescription pharmacies (drug stores part-2) spread throughout Dar es Salaam (Figure 2). The marketing of poor-quality antimalarials was reported in Tanzania [23].

### Results of malaria routine reports

Malaria morbidity and mortality statistics were recorded in the *Infectious Diseases Weekly Report *of all three DMO offices. However, problems with the records were noticed, particularly in Kinondoni District. Over 45% of consultations were diagnosed as clinical malaria in all age groups. According to one source [20], an estimated 1.1 million annual malaria cases were reported in 2000 from 2.2 million outpatient visits to the health facilities, of which half a million were in Kinondoni. However, Stricker [24] found only 320,000 malaria cases reported in the raw dataset of Kinondoni District in 2000. According to the Ilala District Annual Plan 2002, a total of 163,311 malaria cases (under five: 54,853 and five years and above: 108,458) were reported from all health facilities in 2000, while 400,000 cases were estimated in the *Minimum Package of Health-related Management Activities *[20]. The reliability of these data has, therefore, to be questioned.

### School parasitaemia surveys

**Figure 3:**
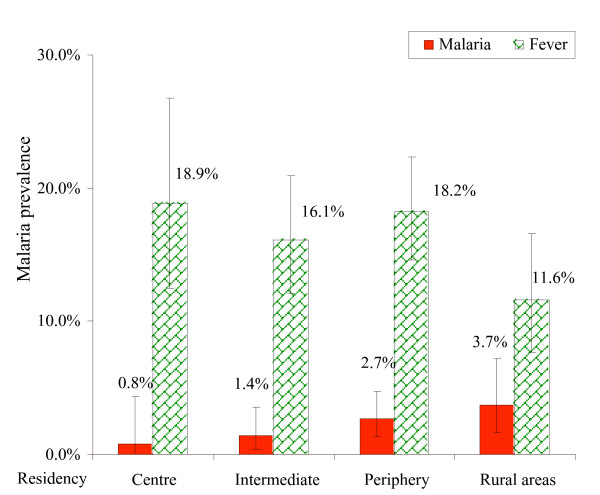
Malaria and fever prevalence rates by residency of schoolchildren. Vertical bars represent 95% CI. School parasitaemia surveys. N = 1054.

*Plasmodium falciparum *was detected in 24 of the 1,054 valid samples (2.1%, 95% CI: 1.2–2.6). Although each of the selected schools had its own catchment area, attending children also came from different areas of the city. The parasitaemia prevalence ranged from 0.8% in the city centre to 3.7% in rural areas, while fever was present in 11.6–18.9% of children (Figure 3). The maximum parasite count was 30,000/μl found in a child in Kijitonyama Ward.

### Health facility-based surveys

**Figure 4:**
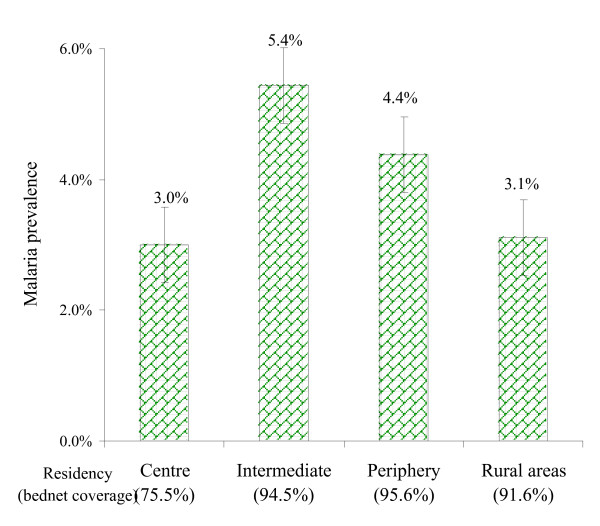
Malaria prevalence rates by residency of patients. Vertical bars represent 95% CI. Health facility-based surveys. N = 1498.

*P. falciparum *was detected in 59 (3.9%) blood films of the 1,498 valid samples (95% CI: 3.0–5.1). Overall, 37/717 (5.2%) fever cases and 22/781 (2.8%) non-fever controls were found positive. The prevalence rate of parasites detected in febrile episodes ranged from 2.0–7.2 % in different age groups, and the rate was lower in the control group, except for infants (Table 1). People living in the intermediate and peri-urban areas of Dar es Salaam had slightly higher parasite prevalence rates than those from the city centre or the rural zone, but the gradient was minimal (Figure 4).

**Figure 5:**
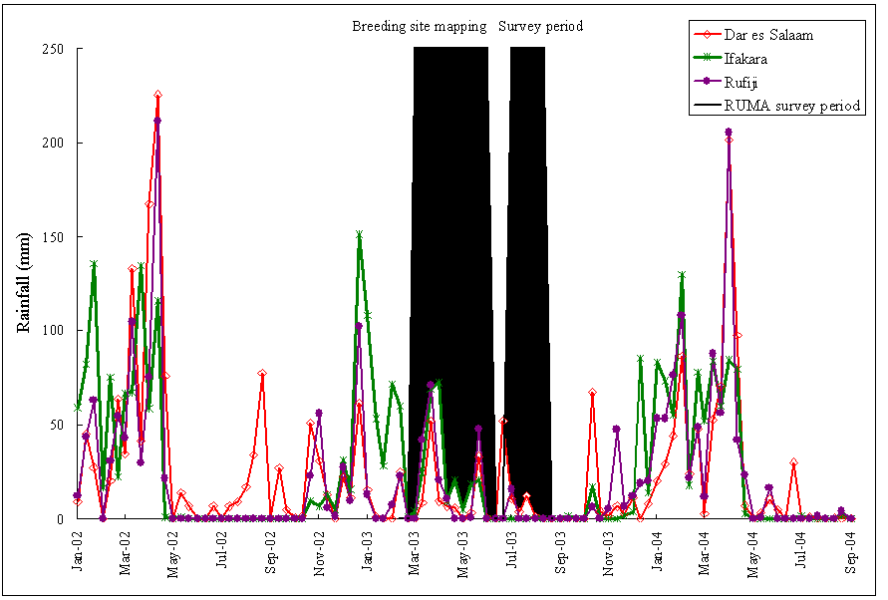


**Table 1:** Malaria infection rates in cases and controls, by age groups. Health facility-based surveys

**Age groups**	**Cases Positive/Total %**	**Controls Positive/Total %**	**OR**	**95% CI**	**P value**
Infants 0–1 year	2/99 (2.0%)	4/116 (3.4%)	0.59	0.07–3.82	0.84
Children 1–5 years	15/213 (7.0%)	8/178 (4.5%)	1.57	0.61–4.14	0.43
Children 6–15 years	7/97 (7.2%)	2/56 (3.6%)	2.02	0.37–14.62	0.61
Adults >15 years	13/308 (4.2%)	8/423 (1.9%)	2.23	0.85–5.96	0.11

The odds ratios (OR) for a malaria infection in fever cases varied between 0.59 and 2.2 in different age groups (Table 1), and the fraction of malaria-attributable fever was extremely low: -0.01, 0.03, 0.04 and 0.02 for the age categories shown in Table 1.

Fevers of two to four days duration were found to be related to a malaria infection compared with fevers of less than two days duration (OR = 1.78, 95% CI = 0.93–3.42, P = 0.08). Further, the risk of having malaria parasitaemia with a fever ≥39°C lasting for two to four days was 7.5 times higher compared to a fever <39°C lasting for two to four days (95% CI = 1.81–28.93, P < 0.05).

### Socio-economic factors and awareness of preventing mosquito biting

A logistic regression model was used to assess the association between the educational level of patients, sources of water supply, having agriculture land or a garden nearby and malaria infections. None of these associations were significant (Table 2). The few people living in a house built with leaves and mud had a higher risk of having malaria compared to those living in a house built with brick and/or concrete (OR = 21.8, 95% CI = 1.29–369.65, P < 0.05). The bednet and insecticide-treated nets (ITN) coverage rates were high in Dar es Salaam (91.8% and 43.1%). Having an ITN seemed to reduce the risk of a malaria infection, while the same level of protection was found for any net, but the result was not significant. The correlation between the amount invested per month in preventing mosquito biting and the risk of malaria was assessed. The OR were 0.54 if the investment ranged from USD 0.5 to 4.9 and 0.69 if the investment ranged from USD 5.0 to 25.0 per month, compared to a smaller amount, but the results were not significant.

**Table 2:** Socio-economic factors and the risk of malaria infection in a logistic regression model. Health facility-based surveys. NA = not available. N = 1449. Significant results are highlighted

**Socio-economic factors**	**% of total**	**Odds-Ratio**	**95% CI**	**P value**
**Education**				
Primary	65.4	1	-	-
Secondary	5.9	0.33	0.04–2.44	>0.05
Superior	1.4	NA	NA	NA
No education	27.3	0.87	0.44–1.72	>0.05

**Housing material**				
Concrete/brick	99.1	1	-	-
**Leaf/mud**	**0.7**	**21.80**	**1.29–369.75**	**<0.05**

**Water supply source**				
Tap water	74.0	1	-	-
Well	24.4	1.19	0.63–2.23	0.6

**Living near a garden or agriculture land**				
No	82.7	1	-	-
Yes	17.3	1.1	0.56–2.16	0.8

**Previous malaria treatment within 30 days with the presence of parasitaemia**				
No		1	-	-
**Yes (≤ 5 years-old)**	**34.5**	**2.84**	**1.33–6.07**	**<0.005**
Yes (>5 years-old)	27.4	0.68	0.27–1.70	>0.05

*Adjusted for the effects of age groups*				

**Bednet usage one night before the survey**				
No	8.2	1	-	-
**Yes**	91.8%	0.6	0.27–1.55	0.3

**ITN ownership**				
No	56.9	1	-	-
**Yes**	**43.1**	**0.6**	**0.34–1.07**	**0.08**

*Adjusted for the effects of different residential areas*				

**Rural exposure within 90 days**				
No		1	-	-
**Yes (≤5 years-old)**	**11.8**	**3.62**	**1.48–8.88**	**<0.05**
**Yes (>5 years-old) **	**13.5**	**2.80**	**1.23–6.37**	**<0.01**

Only 702 out of 1,273 (55.2%) subjects were born in Dar es Salaam, indicating that many were immigrants. Among children under five years of age and those over five years, travelling to rural areas within the preceding 3 months appeared to be a significant risk factor for being infected with *P. falciparum *(Table 2).

In total, 425 subjects declared having had a malaria attack within one month of the survey. Among them, 60.7% were treated by traditional herbs and healers, 27.8% in health facilities, while 5.2% of the sample population only purchased drugs in a pharmacy or a drug outlet. For children under five years of age, there was a significant association between previous malaria treatment and the current presence of parasitaemia (Table 2).

## Discussion and conclusion

The RUMA methodology is based on a cross-sectional study and the results of such a study may be different at another time of year and in different years. The present study was conducted during the dry season of an exceptionally dry year (Figure 5). Therefore, the numbers of larvae breeding sites and clinical malaria cases detected in 2003 may be lower than in years with normal rainfall. These results need to be confirmed in another year and this work is currently ongoing in the frame of the ongoing UMCP.

Weekly epidemiological information was available from each health facility, but the monitoring system did not function adequately. The quality of the routine health statistics was found to be low, and it is not entirely clear how the planning of the patient load takes place, both at city level and for the different health facilities. In addition, the low malaria-attributable fraction of presenting fever episodes (see below) raises ever further the issue of the true malaria burden in this city and the best way to measure it.

The malaria risk mapping was done in conjunction with another project during the rainy season of early 2003 (Figure 5). The breeding sites were identified and mapped over a period of three months. This work gave a good indication of malaria transmission levels in different areas of Dar es Salaam. Without such additional collaboration, mapping of breeding sites would not be possible in the time and budget frame of a RUMA and this component should be dropped from the standard protocol. On the other hand, the mapping of all health facilities is doable if the required list of public and private health service providers can be obtained from the health authorities. In the case of Dar es Salaam the production of such a map was highly appreciated by the authorities and this activity received appropriate support.

The first large post-independence UMCP was carried out between 1986–1994 as a collaboration between the government of Tanzania and the Japanese International Cooperation Agency (JICA). It concluded that in addition to rapid diagnosis and treatment in health facilities, health education, maintenance and cleaning of drains and active participation of the community were of prime importance.

As a result of such activities, the JICA-supported UMCP reduced malaria transmission in Dar es Salaam [9]. Nearly ten years later, the community prevalence of *P. falciparum *in our study was even lower (Table 3). Kisarawe primary school was the only school in the city centre during JICA's intervention, but it no longer existed in 2003, so Mtendeni primary school, about 500 m away, was selected instead. The malaria prevalence was 31% in September, 1988, 7.2% in September, 1991 and then only 2.6% at the end of the UMCP in August, 1995. The parasitaemia rate was only 0.6% in the nearby Mtendeni primary school in July and August, 2003. In the intermediate area, the malaria prevalence was 3% in Sinza and 10% in Kijitonyama Kisarawe primary school during September-December, 1994 [8], compared with Mwenge (0.9%) and Kijitonyama Kisarawe primary school (2.1%) in 2003. Due to administrative problem, Sinza was replaced by Mwenge primary school, around 1 km away. In the periphery, the malaria prevalence in Kigamboni primary school was 41% in 1988, 14.7% in September, 1991 and then only 9.3% in 1995, compared to 3.0% in Ufukoni primary school in August, 2003. Ufukoni is around 200 meters to Kigamboni primary school. In the surrounding rural area, Buza primary school was about five km closer to the urban zone than Chamzi primary school which was selected in the JICA study. The malaria prevalence was 28% in Chamzi in September-December 1994 and 3.8% in Buza primary school in August 2003. Hence, our results showed a lower level of endemicity compared to the 1990s. Results could be extremely different during and at the end of rainy season or in different year. A household survey was going on in 18 wards in Dar es Salaam (6 wards in each district) from May, 2004. The unpublished result showed that the malaria prevalence varied widely in different communities and different seasons: it ranged from 1.5–44.6% in 2004 and 3.7–50.9% in 2005 (G. Killeen, personal communication).

**Table 3:** Malaria prevalence in primary schools in Dar es Salaam in the JICA-UMCP study between 1988 and 1995 and the RUMA study in 2003, by geographical location

**Malaria prevalence**	**Central**		**Intermediate**			**Periphery**		**Rural area**	
Primary schools	Kisarawe	Mtendeni	Sinza	Kijitonyama	Mwenge	Kigamboni	Ufukoni	Chamzi	Buza
Sep-88	31.0%	-	-	-	-	41.0%	-	-	-
Aug-89	11.7%	-	-	-	-	7.3%	-	-	-
Aug-90	9.5%	-	-	-	-	13.5%	-	-	-
Sep-91	7.2%	-	-	-	-	14.7%	-	-	-
Jul-92	0.9%	-	-	-	-	10.6%	-	-	-
Sep-94	6.5%	-	3.0%	10.0%	-	14.1%	-	28.0%	-
Aug-95	2.6%	-	-	-	-	9.3%	-	-	-
Jul-Aug 03	-	0.6%	-	2.1%	0.9%	-	3.3%	-	3.8%

It is difficult to assess whether this is a lasting trend, brought about, for example, by the high level of ITNs use or increasing urbanization, or whether this was the result of an especially dry season. In any case, the population of Dar es Salaam was well aware of malaria prevention, especially the use of ITNs and this was also reported by other studies [24, 25, 26].

In 1988, 20% of all persons of working age in Dar es Salaam were involved in some ways in urban agriculture [27]. A recent study confirmed that urban agriculture is widespread in Dar es Salaam [28]. Unfortunately, these activities create a suitable breeding ground for malaria vectors. Sattler *et al*. [16] identified more than 400 *Anopheles sp. *breeding sites in central Dar es Salaam, which was surprising given the low level of endemicity. An ongoing survey showed that mosquito landing rates per person per night in Dar es Salaam were very low, implying that larvae and pupae of *Anopheles sp. *are perhaps unable to develop to adult mosquitoes (Y. Geissbühler and G. Killeen, personal communication).

The fractions of malaria-attributable fevers in health facilities were low in all age groups during the time of the present survey, suggesting that patients presenting at health facilities with fever were much more prone to suffer from other diseases than malaria. Less than 5% of all fever-related consultations in Dar es Salaam were likely to be due to malaria during the dry season of 2003 and this has important implications for fever case management. On the one hand this leads to a substantial number of unnecessary treatments, a problem made much more serious with the forthcoming introduction of the more expensive artemisinins-based combination therapy. On the other hand, over-diagnosing malaria patients may also distract from other causes of fever, some of which may be dangerous to the patient. Meanwhile, it is urgent to estimate the fractions of malaria-attributable fevers during the rainy season and to review carefully the procedures for malaria diagnosis in health facilities. In a second step, revised guidelines for the management of fever cases may need to be considered.

## Authors' contributions

SW participated in the design of the study, conducted the field work, analysed and interpreted data, drafted and revised the manuscript. CL conceived the study, coordinated the field work, interpreted the data and revised the manuscript. DM was the key local contact person; he coordinated and supervised the field activities. TM was in charge of laboratory work and quality control of slides. LM and GM participated in the data collection, the entry of data and the mapping of health facilities. MT participated in the conception of the work and revised it critically at different stages.
